# Supplementary material for: Preventing excessive autophagy protects from the pathology of mtDNA mutations in Drosophila melanogaster
Source: Nat Commun. 2024 Dec 23;15:10719. doi: 10.1038/s41467-024-55559-2 (PMC11666730; doi:10.1038/s41467-024-55559-2)
Supplement: Supplementary file 1 — Supplementary Information [file 41467_2024_55559_MOESM1_ESM.pdf]

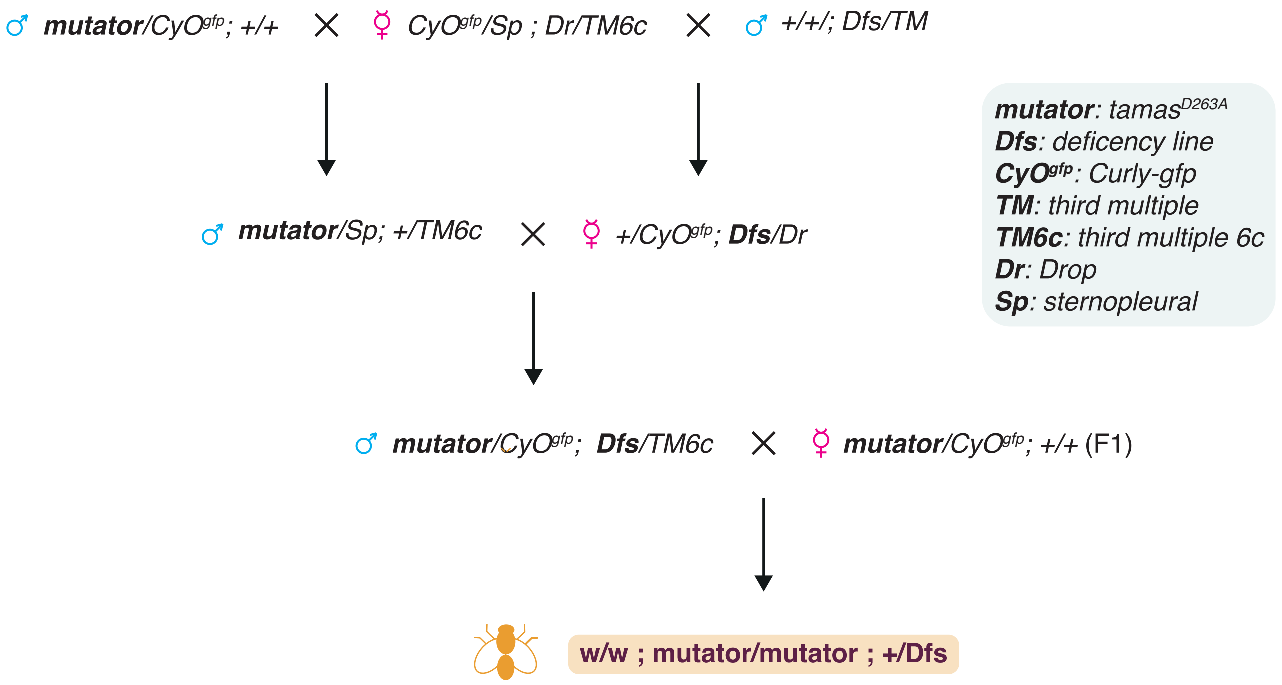

Supplementary Figure 1. Scheme of genetic crosses for deficiency screen

Crossing scheme to generate heterozygous deficiency lines on a homozygous *tamas*^{D263A} background. Crosses were designed to ensure minimal maternal inheritance of the *tamas*^{D263A} allele. The screen was repeated with the mutator/-glossy;+/+ balancer to control for the genetic background.

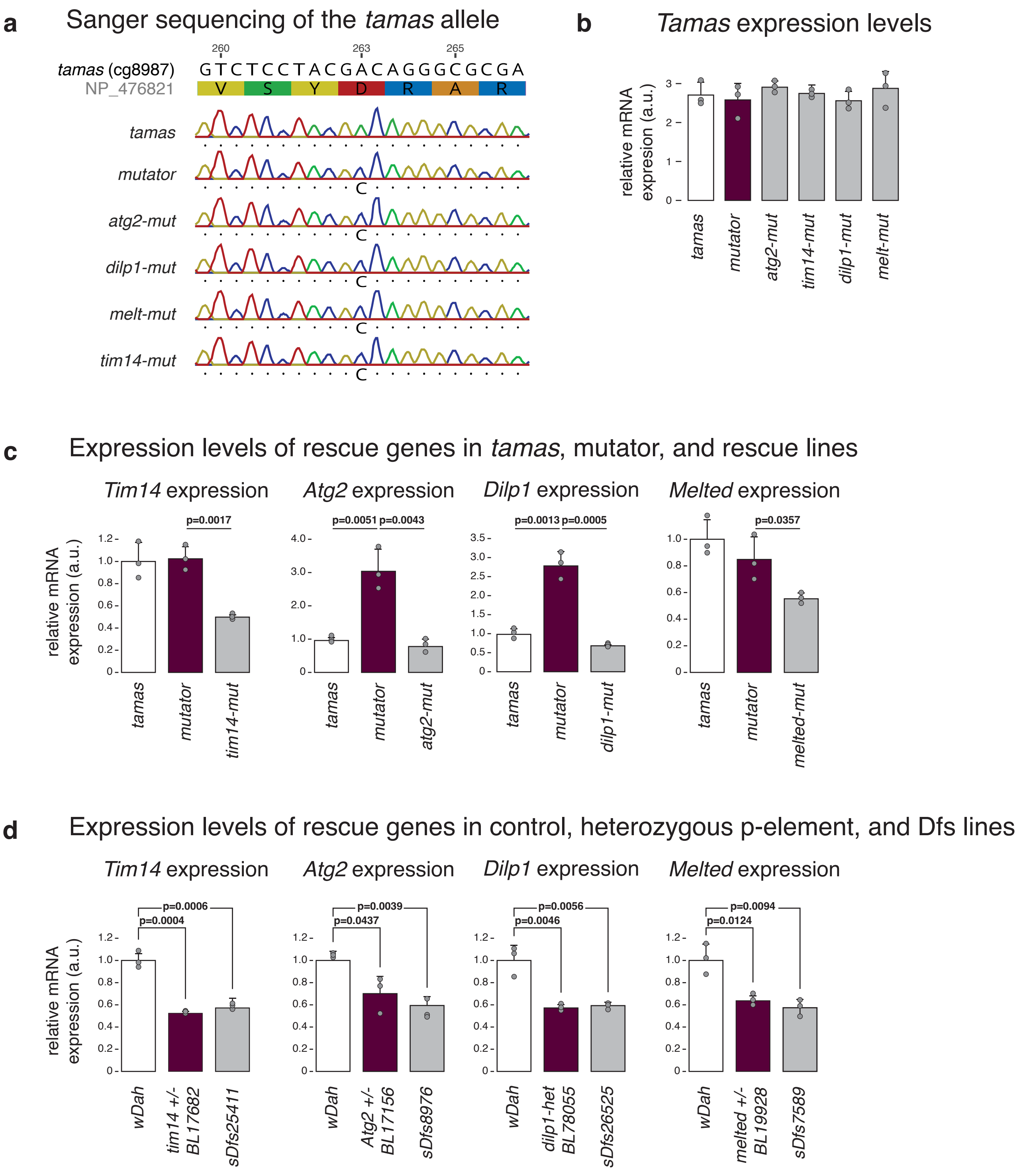

Supplementary Figure 2. Validation of the mitorescue lines

(**a**) Sanger sequencing of the tamas allele, showing the c.788A>C substitution, leading to a p263D>A mutation. (**b**) *Relative tamas* mRNA expression levels in 3rd instar larvae. In control, mutator, or mitorescue larvae. Expression levels were normalised to *rp49* expression. Mean value of 3 biological replica per genotype with 10 larvae each. (**c**) Relative expression levels of the targeted mitorescue genes in control (*tamas*), homozygous mutator, and the respective rescued 3rd instar larvae in a wild-type tamas background. Mean value of 3 biological replica per genotype with 10 larvae each. Two-tailed Student's T-test with equal variance was used with mutator against other genotypes. (**d**) Relative expression levels of the targeted mitorescue genes in control (*wDah*), heterozygous (p-element) mutant (Bloomington reference numbers are shown), or heterozygous small deficiency lines (deficiency reference codes are shown) of 3rd instar larvae in a wild-type *wDah* background. Mean value of 3 biological replica per genotype with 10 larvae each. Two-tailed Student's T-test with equal variance was used with control (*wDah*) against other genotypes. P values <0.05 are shown in bold. Error bars represent Standard deviation. Source data are provided in the supplementary figure source data file.

a *Tom40* expression levels

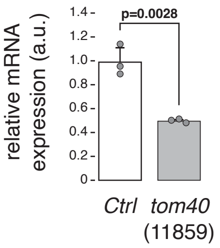

b Simplified nutrient sensing signalling pathway

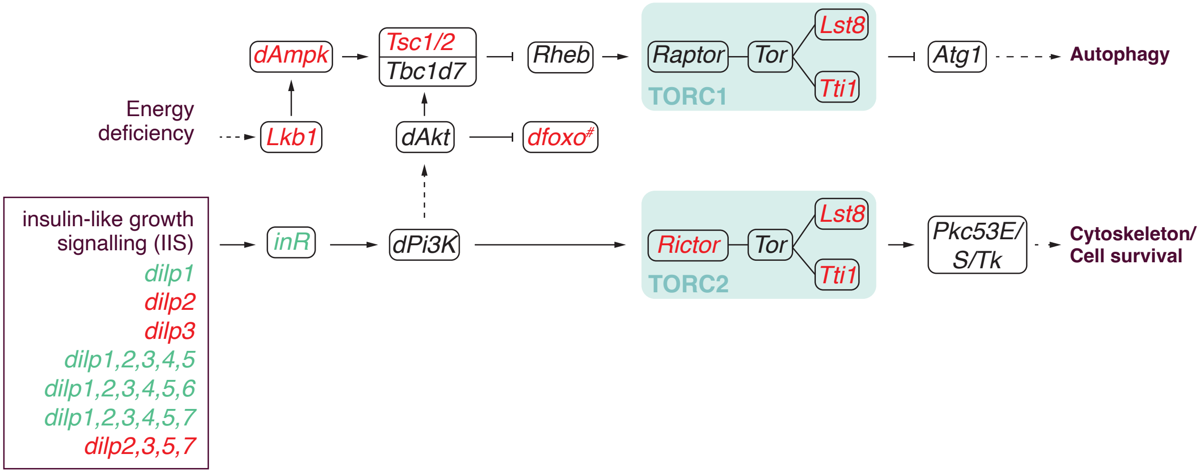

c mtDNA levels in *dilp-mut* larvae

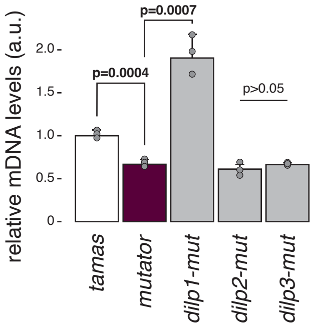

d mtDNA mutation load (RMC)

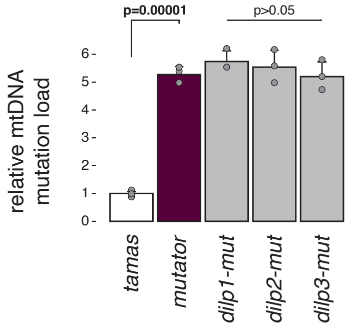

Supplementary Figure 3. Testing the *Drosophila* insulin signalling pathway for rescue capability

(**a**) Relative tom40 expression levels in line BL11859 as determined by qRT-PCR. *Rp49* was used as loading control (N=3 biological replicates with 3 technical replicates). (**b**) Simplified diagram of insulin and TOR signalling pathways adapted to *Drosophila*. Tested *Drosophila* lines are shown in colour with rescuing heterozygous KO combinations shown in green. Insulin Ligands (DILPs). # No double heterozygous KO flies hatched for *dFoxo* (*+/Polg*$^{exo-}$ *; +/dfoxo*$^{-}$). Only genes with the green colour can rescue the mutator larva lethality. (**c**) Relative mDNA levels in 3rd instar larvae as determined by RT-PCR. *Rp49* was used for normalisation. Mean value of 3 biologically independent samples with 3 technical replica are shown. (**d**) Relative mtDNA mutation load as determined by random mutation capture assay (RMC). Total DNA from 10 3rd instar larvae were digested by TaqI endonuclease, followed by RT-PCR. Results are shown as mean relative to *tamas*, with 3 biologically independent samples per genotype with 3 technical replicates.

Two-tailed Student's T-test with equal variance was used with mutator against other genotypes. P values <0.05 are shown in bold. Error bars represent Standard deviation. Source data are provided in the supplementary figure source data file.

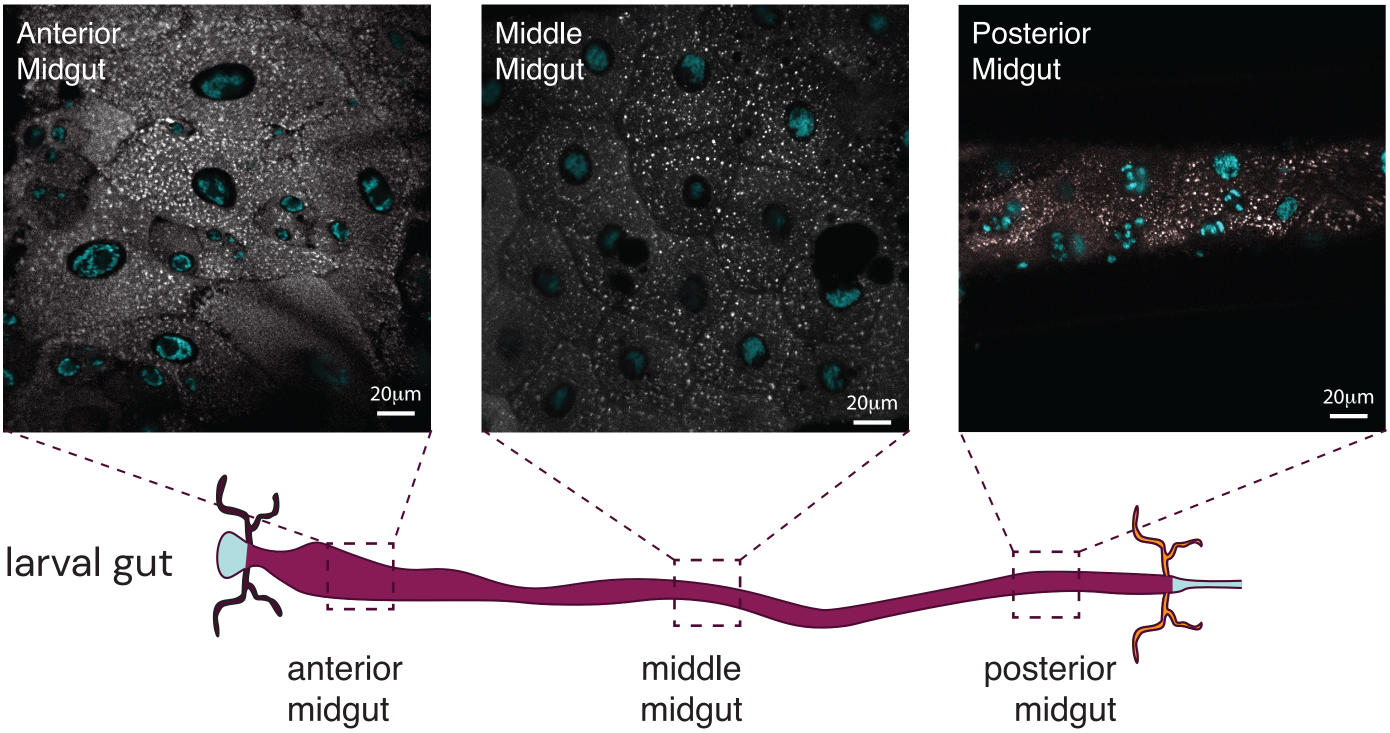

Supplementary Figure 4. Anatomy of the *Drosophila* gut
Anterior midgut, middle midgut, and posterior midgut were stained with TRME staining to demontrate the significantly different cell morphology in the different gut regions. Magnification as indicated.

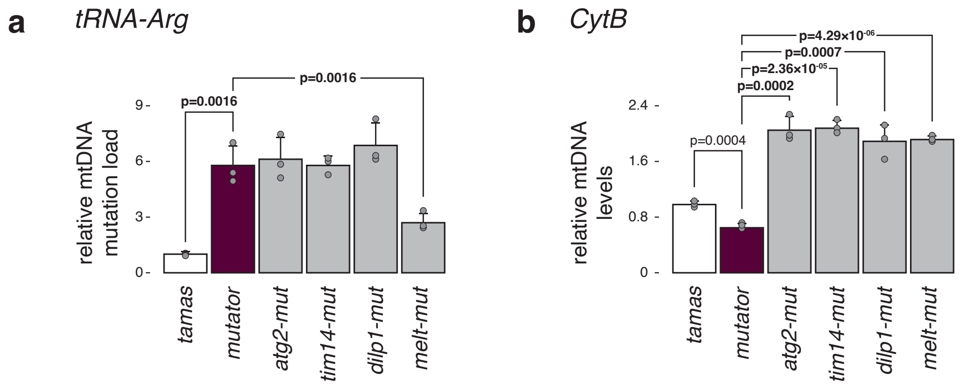

Supplementary Figure 5. MtDNA levels and mtDNA mutation load quantification
(**a**) Relative mtDNA mutation load determined by random mutation capture assay (RMC). Total DNA from 10 3rd instar larvae were digested by TaqI endonuclease, followed by RT-PCR. Results are shown as mean relative to *tamas*, with 3 biologically independent samples per genotype with 3 technical replica. (**b**) Relative mDNA levels in 3rd instar larvae as determined by RT-PCR. *Rp49* was used for normalisation. Mean value of 3 biologically independent samples with 3 technical replica are shown.
Two-tailed Student's T-test with equal variance was used with mutator against other genotypes. P values <0.05 are shown in bold. Error bars represent Standard deviation. Source data are provided in the supplementary figure source data file.

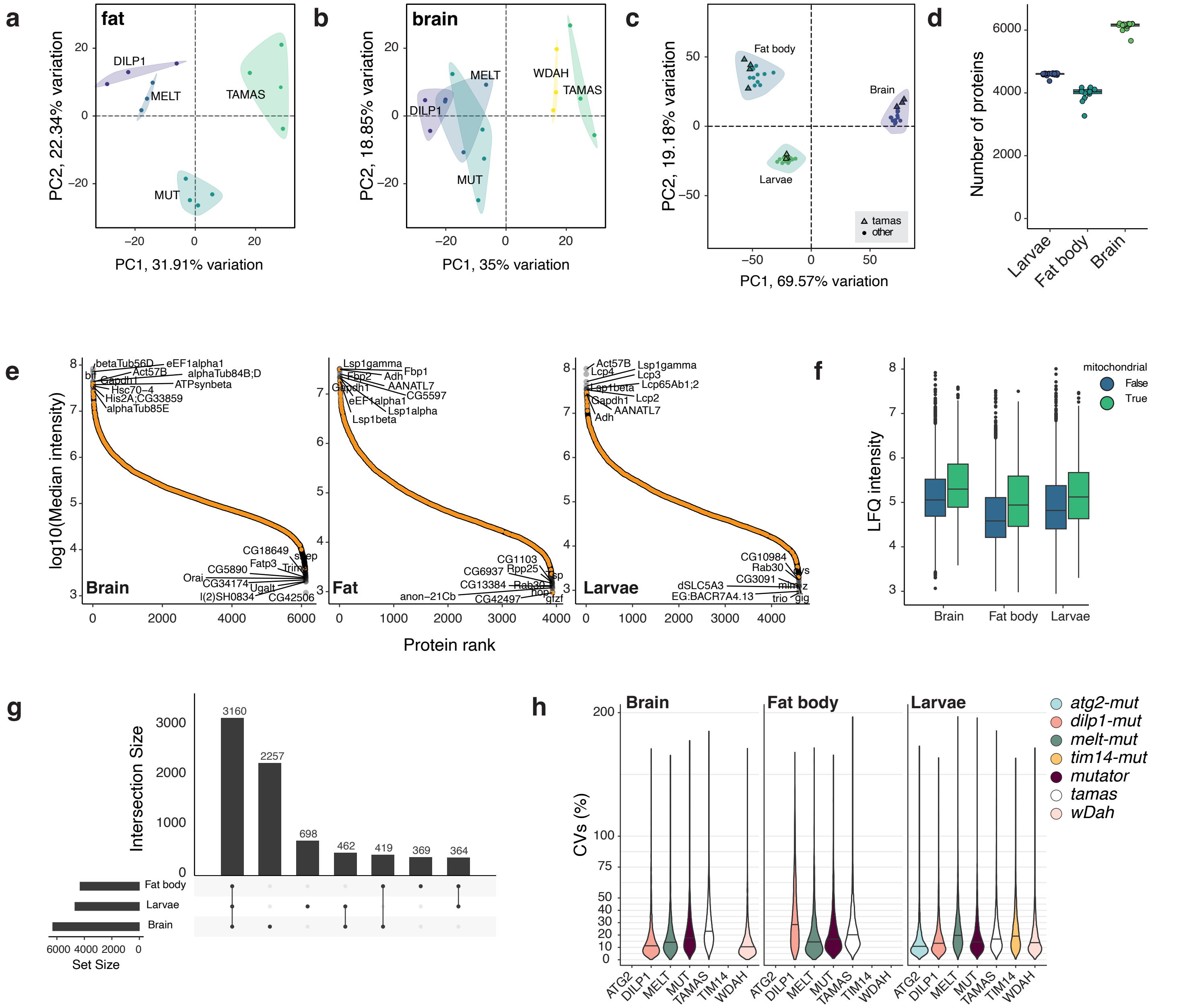

Supplementary Figure 6: Overview of proteomics data with quality control in total larvae, isolated fat bodies, and brain.

(**a**) Principle component analysis of dissected fat body and (**b**) brain tissue. Each dot is one sample. (**c**) Grouping of tissue-specific proteomes, including all measured genotypes. (**d**) Number of proteins per sample. Each dot is one sample. (**e**) Abundance range curves of all tissue types, showing the median protein intensities across genotypes. The top and lowest 10 proteins are marked. Orange dots denote mitochondrial proteins. (**f**) Intensities of mitochondrial and non-mitochondrial proteins by tissue type. The median for all genotypes is plotted. The box is the first and third quartile, with thick line as median. Whiskers are ±1.5 interquartile range, and additional black dots are outliers. (**g**) Unique and shared proteins across three tissues. The set size is the total number of unique proteins. (**h**) Violin plot of coefficients of variation (CVs) per protein as percentage per tissue and genotype. The colour coding is genotype specific. The thick line is the median.

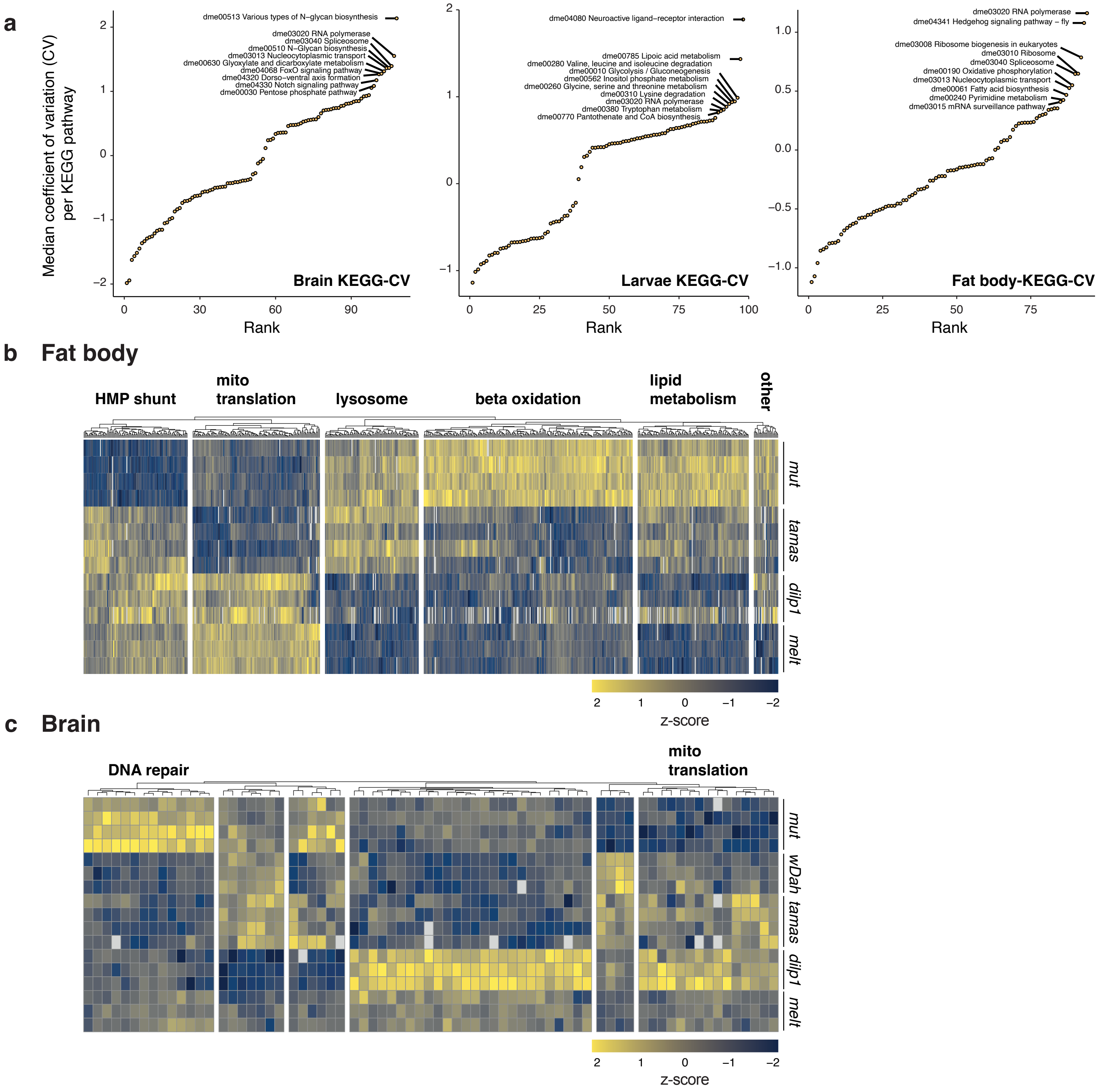

Supplementary Figure 7: Functional enrichments in proteomics data.
(**a**) Coefficient of variation (CV) as percentage across normalised enrichment scores of all rescue genotypes versus mutator, facetted by tissue type. The underlying functional library is the Kyoto Encyclopaedia of Genes and Genomes. Top ten variable categories are annotated. (**b**) Expression of significant different proteins (ANOVA, FDR < 0.01) as z-scores in fat body, and (**c**) brain. Functional categories are significantly enriched gene ontologies (Fisher's exact test, FDR < 0.05). Each column is one replicate.

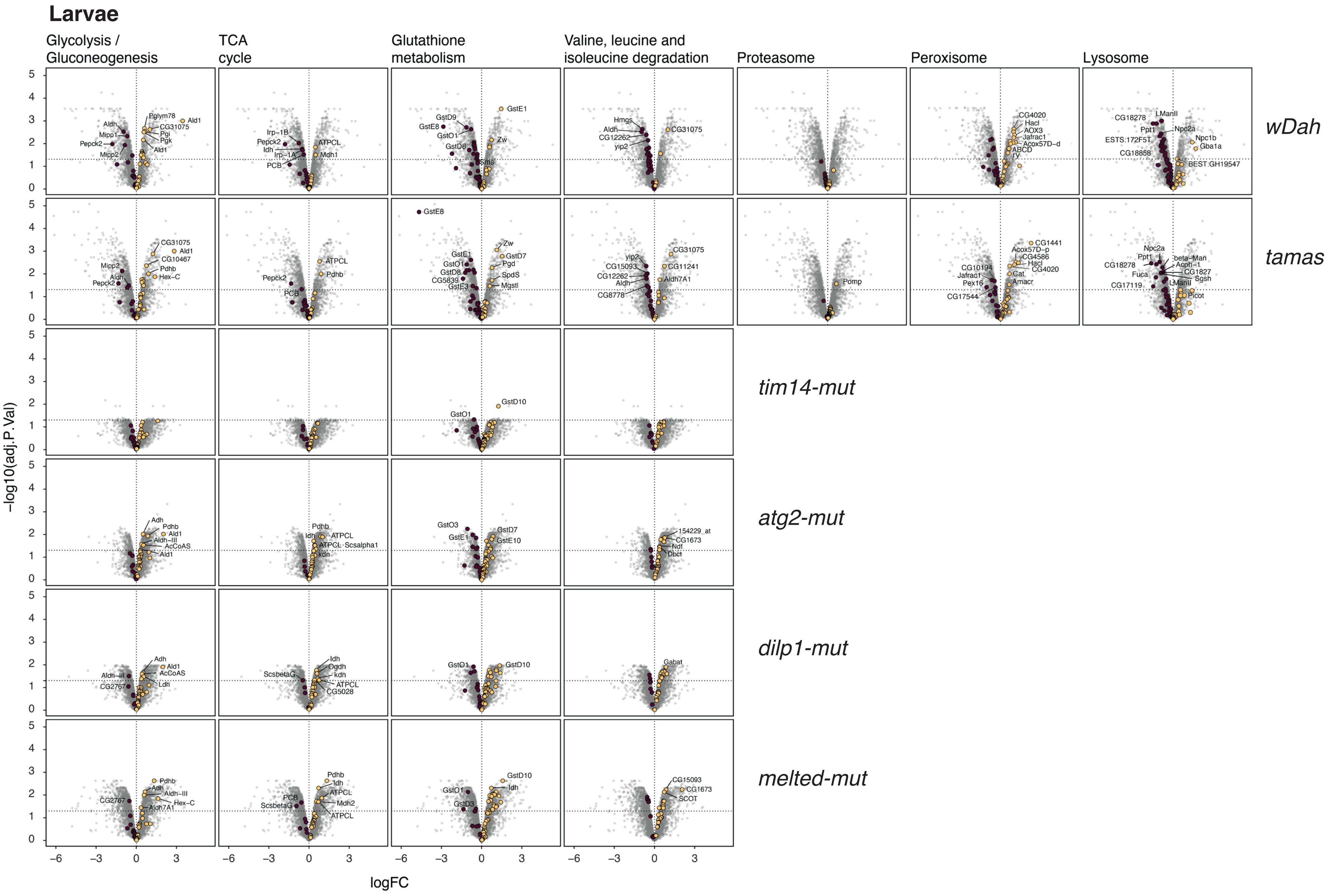

Supplementary Figure 8: Volcano plots showing functional enrichment of selected KEGG pathways.
Colour-coding refers to down- (plum) or upregulation (yellow) of indicated genotype with mutator larvae. Selected significant hits (FDR < 0.05) are annotated.

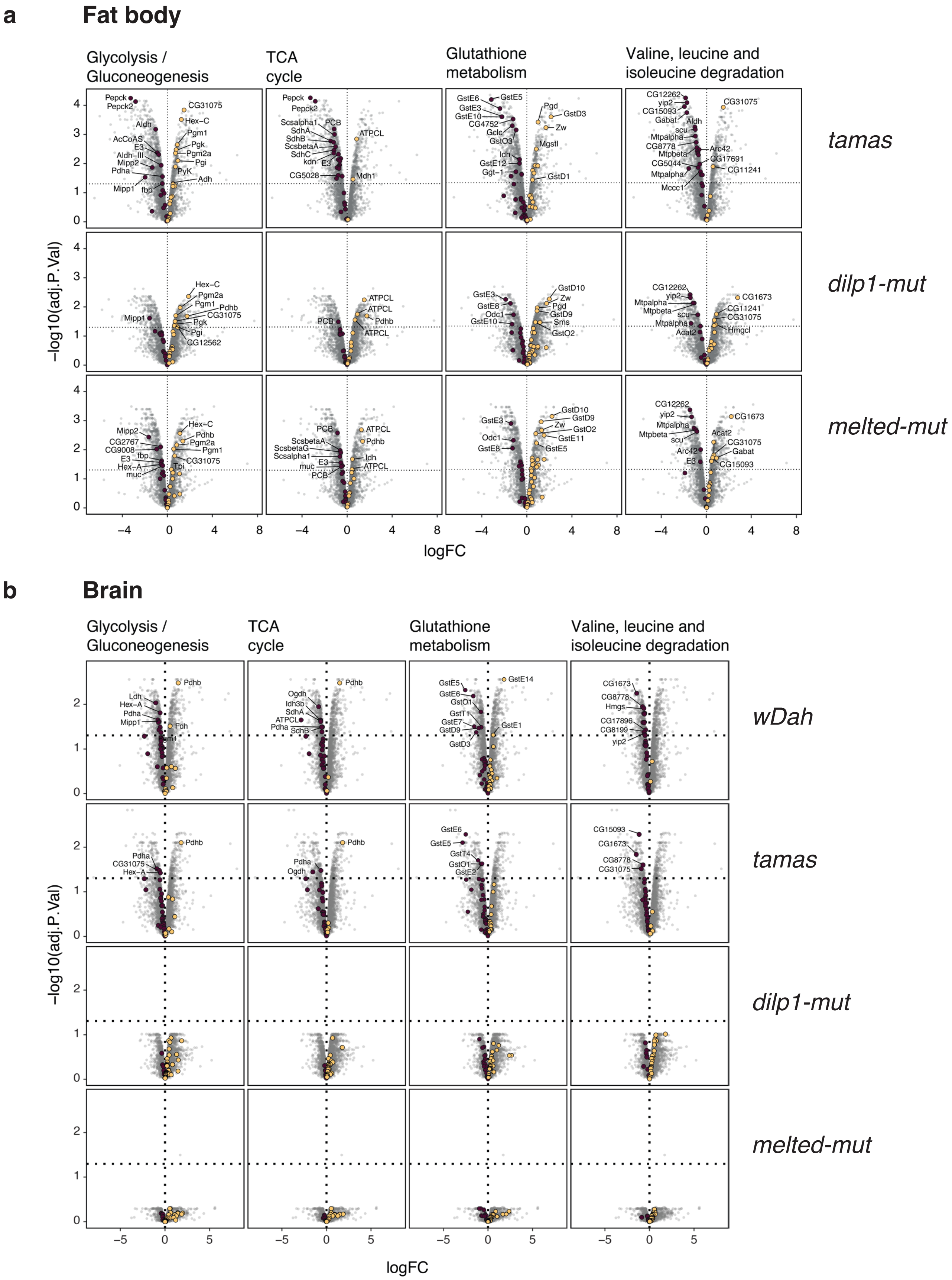

Supplementary Figure 9: Functional enrichment of selected KEGG pathways.
Functional enrichment of selected KEGG pathways in (**a**) fat body and (**b**) brain. The colour-coding refers to down- or upregulation on top of a Volcano plot comparing the indicated genotype with mutator larvae. Significant hits (FDR < 0.05) are annotated.

a

b

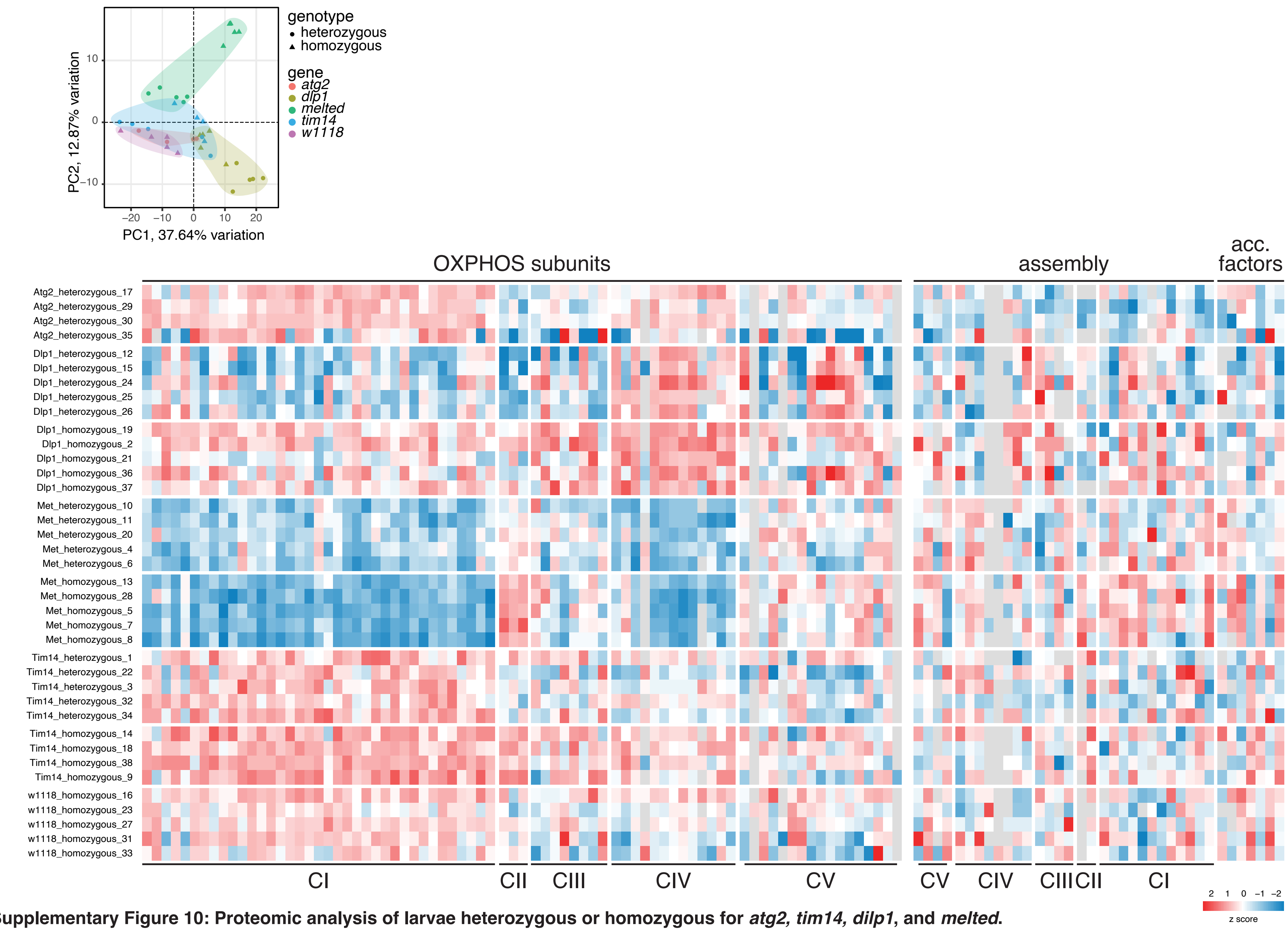

Supplementary Figure 10: Proteomic analysis of larvae heterozygous or homozygous for *atg2, tim14, dilp1*, and *melted*.
(**a**) Principal components 1 and 2 of larval proteomes. Each dot is one sample. Colour overlay groups genotypes. (**b**) LogFC of OXPHOS, assembly, and accessory subunits relative to w[1118] control larvae (n = 4).

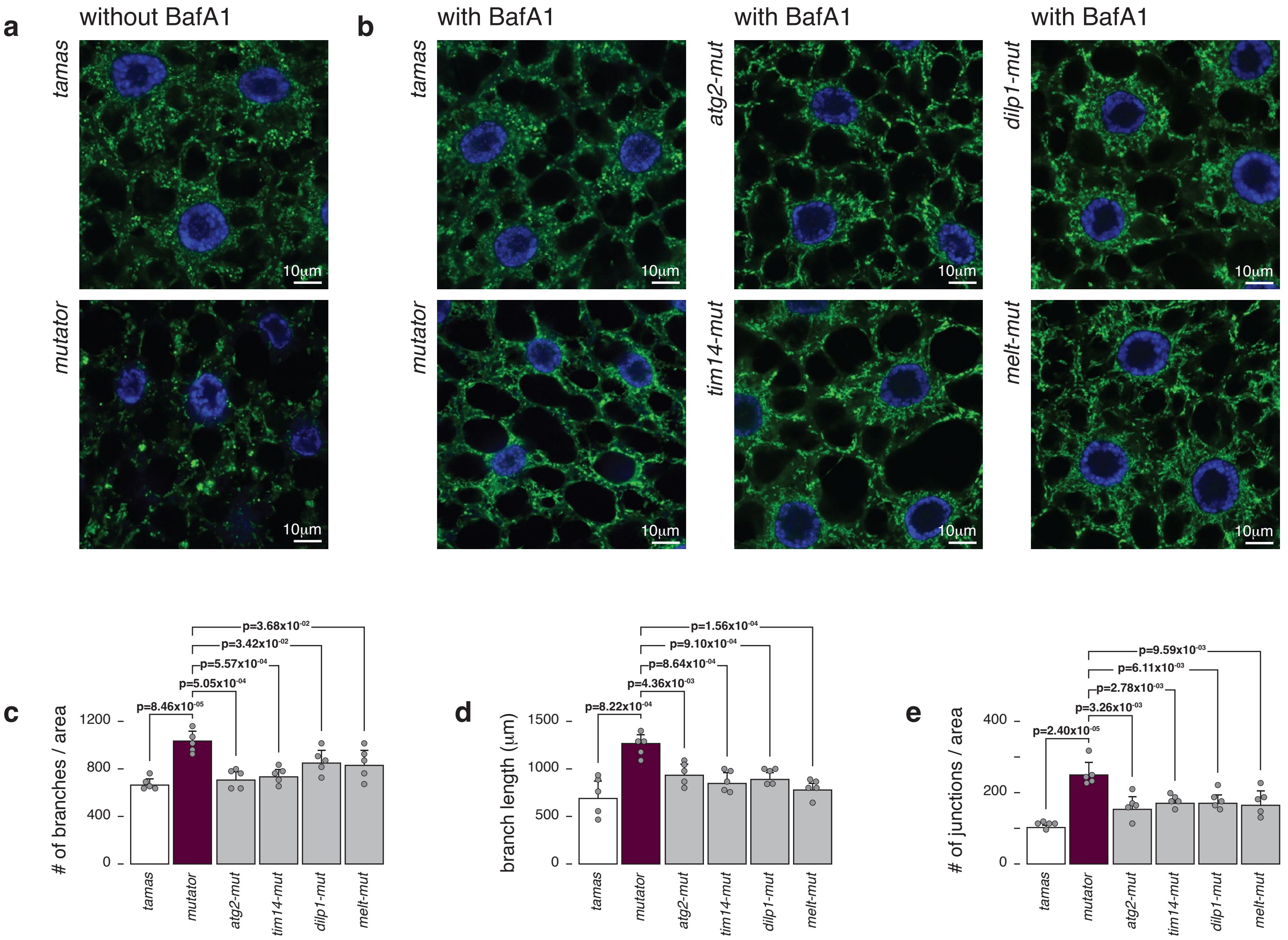

Supplementary Figure 11: Mitochondrial morphology in larvae fat bodies.

(**a**) Representative confocal image of fat bodies of control (*tamas*) and *Polg*$^{exo-}$ (*mutator*) larvae at 4 days after egg laying, showing mitochondria in green (α-ATP-synthase) and nucleus in blue (DAPI). (Scale bar = 10 μm).(**b**) Representative confocal image of fat bodies treated with BafA1 of control (*tamas*), *Polg*$^{exo-}$ (*mutator*), and rescue lines (as indicated) larvae at 4 days after egg laying, showing mitochondria in green (α-ATP-synthase) and nucleus in blue (DAPI). (Scale bar = 10 μm). (**c**) Number of mitochondrial branches per area (81μm^2) from images represented in (b). (N=5 biological replicates) (**d**) Mitochondrial branch length calculated from images represented in (b). (N=5 biological replicates) (**e**) Number of mitochondrial junctions per area (81μm^2) from images represented in (b). (N=5 biological replicates)

Two-tailed Student's T-test with equal variance was used with mutator against other genotypes. P values <0.05 are shown in bold. Error bars represent Standard deviation. Source data are provided in the supplementary figure source data file.

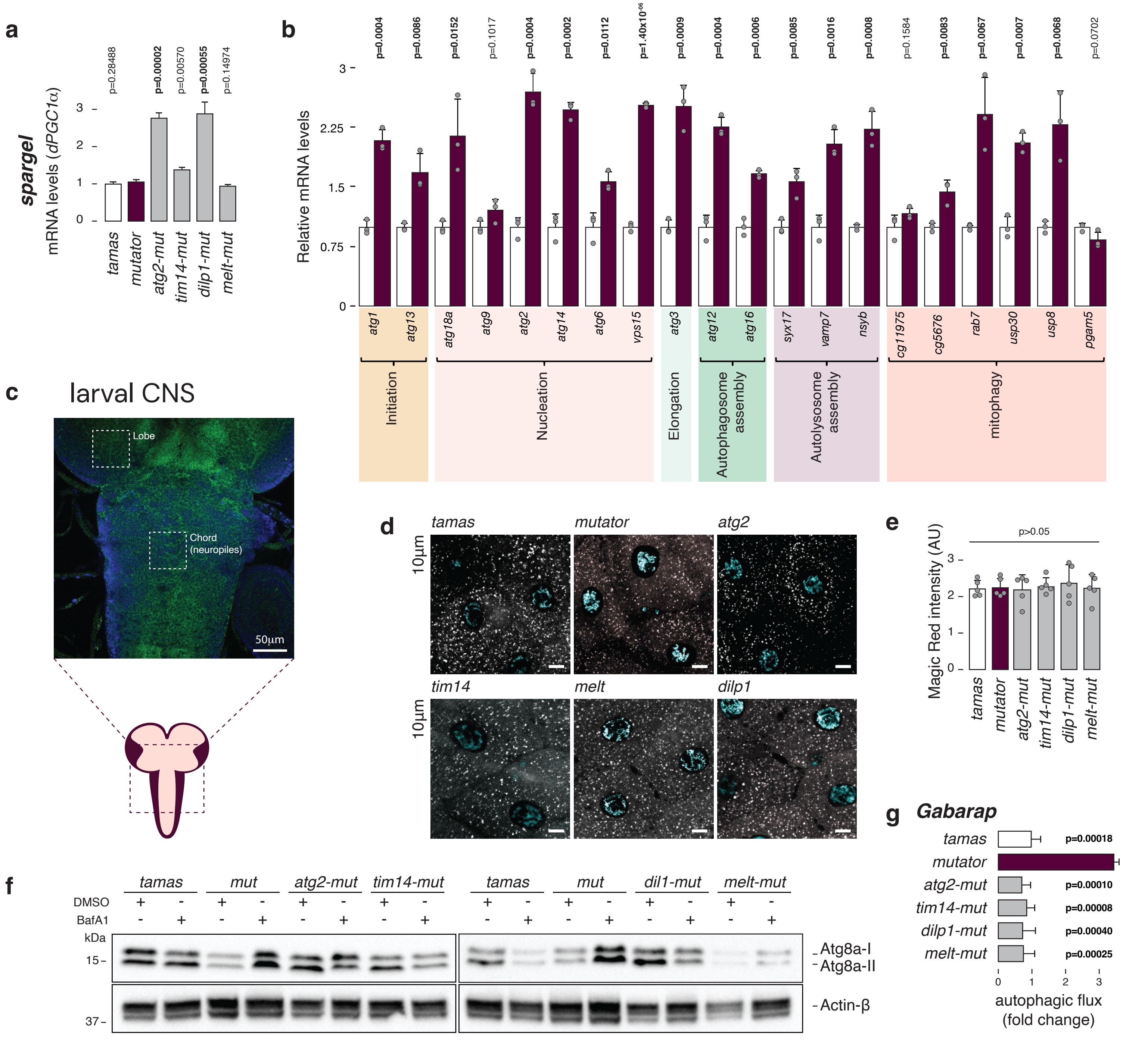

Supplementary Figure 12. Increased autophagic flux in mutator larvae

(**a**) Relative mRNA expression levels of *spargel* (*dPGC1α*) in 3rd instar larvae as determined by qRT-PCR Levels were normalised to *rp49* mRNA. Mean relative to *tamas*, with 3 biologically independent samples per genotype with 3 technical replica. (**b**) Relative mRNA level of autophagy and mitophagy genes are measured in 3rd instar tamas and mutator larvae. Mean relative to *tamas*, with 3 biologically independent samples per genotype with 3 technical replica. (**c**) Representative zoomed-out confocal image of the *Drosophila* central nervous system (CNS) decorated with antibodies against (α-ATP-synthase (green) and the nucleus with DAPI (blue). (**d**) Representative zoomed-out confocal image of the *Drosophila* larvae digestive system stained with MagicRed (grey) and Hoechst (cyan). (**e**) Quantification of (d). (**f**) Western blot analysis on Atg8a lipidation in *tamas*, mutator, and rescue flies. GABARAP (anti-Atg8a) recognises lipidated Atg8a-II (lower band) and non-lipidated Atg8a-I (upper band). Each replica contains 10 3rd instar larvae, incubated with 400 μM of BafA1 or DMSO prior to Western blots. Actin-beta was used as the loading control. (**g**) Quantification of Atg8a-II versus actin of (f). N=3 biological replicates. Two-tailed Student's T-test with equal variance was used with mutator against other genotypes in (a), (e), and (g), and against *tamas* in (b). P values <0.05 are shown in bold. Error bars represent Standard deviation. Source data are provided in the supplementary figure source data file.

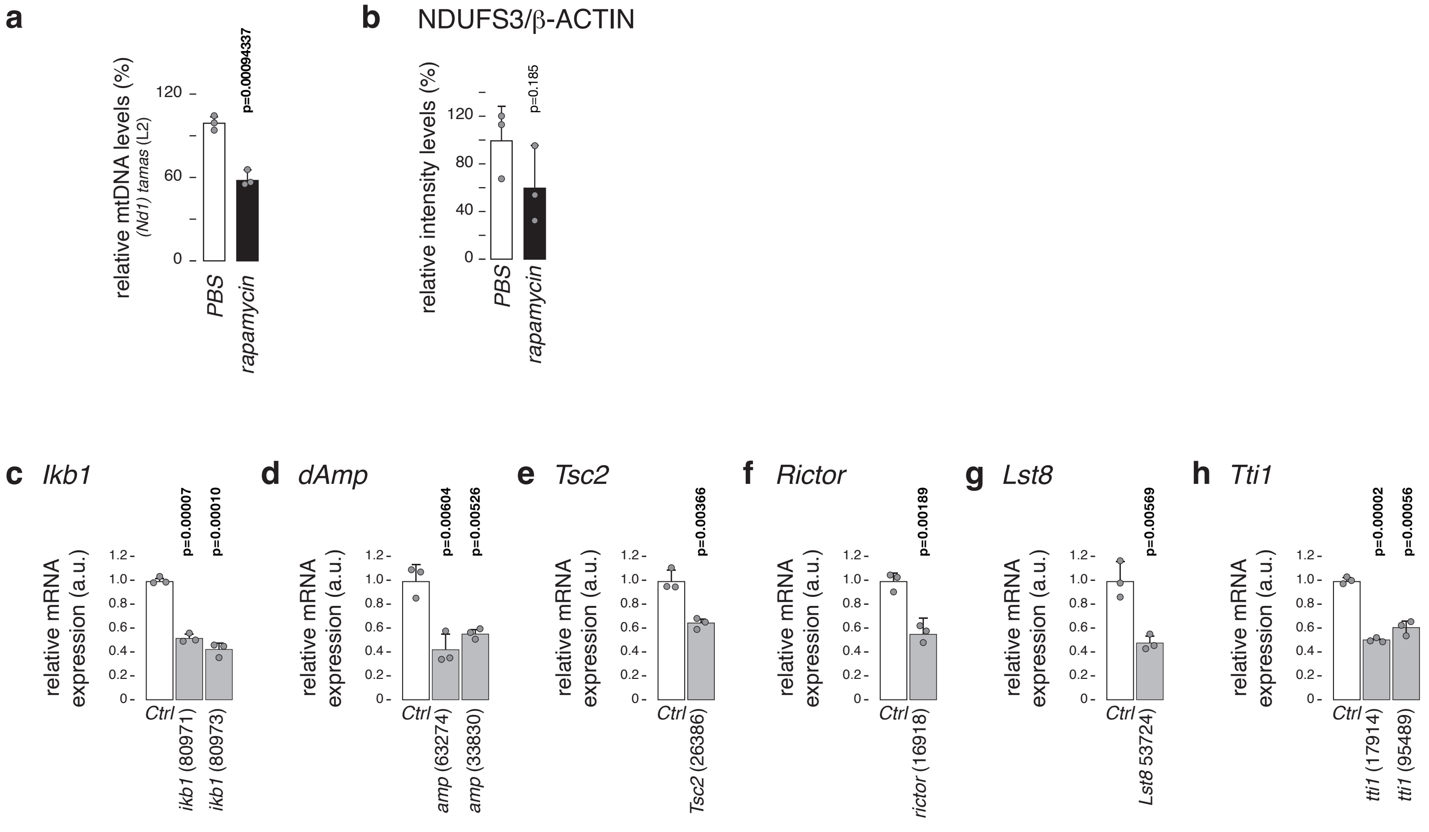

Supplementary Figure 13. Rapamycin treatment and mRNA expression levels of TOR pathway genes.
(**a**) Relative mtDNA levels of control larvae treated with PBS or rapamycin. N=3 biological replicates with 3 technical replicates (**b**) Relative protein levels of NDUFS3 as determined by Western blot analysis in control larvae treated with PBS or rapamycin. β-actin was used as loading control. N=3 biological replicates. (**c-h**) Relative mRNA expression levels in 3rd instar larvae control (Ctrl) and heterozygous mutant lines for (c) *Ikb1*, (d) *dAmp* , (e) *Tsc2*, (f) *Rictor*, (g) *Lst8*, (h) *Tti1*. 10 larvae per sample. Bloomington strain numbers are shown. N=3 biological replicates with 3 technical replicates.
Two-tailed Student's T-test with equal variance was used with control (for c-h) or mutator (for i,j) against other genotypes. P values <0.05 are shown in bold. Error bars represent Standard deviation. Source data are provided in the supplementary figure source data file.
