# Supplementary material for: Preventing excessive autophagy protects from the pathology of mtDNA mutations in Drosophila melanogaster
Source: Nat Commun. 2024 Dec 23;15:10719. doi: 10.1038/s41467-024-55559-2 (PMC11666730; doi:10.1038/s41467-024-55559-2)
Supplement: Supplementary file 2 — Description of Additional Supplementary Files [file 41467_2024_55559_MOESM2_ESM.pdf]

Description of Additional Supplementary Files

File name: Supplementary Data 1
Description: List of fly lines used in this study, containing their order number and survival status on a homozygous mtDNA mutator background.

File name: Supplementary Data 2
Description: List of mtDNA mutations identified by cloning and sequencing.

File name: Supplementary Data 3
Description: Processed proteome data from brain, fat, and total larvae from control, mutator and rescue-mutator larvae.

File name: Supplementary Data 4
Description: Processed proteome data from larvae of heterozygous and homozygous for the identified atg2, melted, tim14, and dilp1.

File name: Supplementary Data 5
Description: List of primers used in this study.

File name: Supplementary Data 6
Description: List of Antibodies used in this study.

File name: Supplementary Movie 1
Description: Movies of larvae movement.
