# Supplementary material for: Preventing excessive autophagy protects from the pathology of mtDNA mutations in Drosophila melanogaster
Source: Nat Commun. 2024 Dec 23;15:10719. doi: 10.1038/s41467-024-55559-2 (PMC11666730; doi:10.1038/s41467-024-55559-2)
Supplement: Supplementary file 5 — Reporting Summary [file 41467_2024_55559_MOESM5_ESM.pdf]

nature portfolio

Corresponding author(s): Christoph Freyer
Anna Wredenberg

Last updated by author(s): Dec 1, 2024

Reporting Summary

Nature Portfolio wishes to improve the reproducibility of the work that we publish. This form provides structure for consistency and transparency in reporting. For further information on Nature Portfolio policies, see our Editorial Policies and the Editorial Policy Checklist.

Statistics

For all statistical analyses, confirm that the following items are present in the figure legend, table legend, main text, or Methods section.

n/a	Confirmed	
☐	☒	The exact sample size (*n*) for each experimental group/condition, given as a discrete number and unit of measurement
☐	☒	A statement on whether measurements were taken from distinct samples or whether the same sample was measured repeatedly
☐	☒	The statistical test(s) used AND whether they are one- or two-sided<br>*Only common tests should be described solely by name; describe more complex techniques in the Methods section.*
☒	☐	A description of all covariates tested
☒	☐	A description of any assumptions or corrections, such as tests of normality and adjustment for multiple comparisons
☐	☒	A full description of the statistical parameters including central tendency (e.g. means) or other basic estimates (e.g. regression coefficient) AND variation (e.g. standard deviation) or associated estimates of uncertainty (e.g. confidence intervals)
☐	☒	For null hypothesis testing, the test statistic (e.g. *F*, *t*, *r*) with confidence intervals, effect sizes, degrees of freedom and *P* value noted<br>*Give P values as exact values whenever suitable.*
☒	☐	For Bayesian analysis, information on the choice of priors and Markov chain Monte Carlo settings
☒	☐	For hierarchical and complex designs, identification of the appropriate level for tests and full reporting of outcomes
☐	☒	Estimates of effect sizes (e.g. Cohen's *d*, Pearson's *r*), indicating how they were calculated

Our web collection on statistics for biologists contains articles on many of the points above.

Software and code

Policy information about availability of computer code

Data collection	Proteomic data acquisition was carried out in DIA mode with a scan range of 300-1300 m/z and a resolution of 120,000.
Data analysis	Fragment ions were analyzed in 40 DIA windows at a resolution of 30,000, while the AGC was maintained at 1e6. DIA raw files were processed using Spectronaut (v16.3) using default settings. Protein intensities were further analysed in R version 4.3.1, using limma linear modelling differential expression analysis for statistical testing.<br>Geneious Prime (v2024) was used for sequence alignments.<br>Adobe Illustrator (v2025) was used for figure preparation<br>Adobe Lightroom (v2023) as used for image preparation<br>Image J (v 1.54e 4) was used for image analysis

For manuscripts utilizing custom algorithms or software that are central to the research but not yet described in published literature, software must be made available to editors and reviewers. We strongly encourage code deposition in a community repository (e.g. GitHub). See the Nature Portfolio guidelines for submitting code & software for further information.

Data

Policy information about availability of data

All manuscripts must include a data availability statement. This statement should provide the following information, where applicable:

- Accession codes, unique identifiers, or web links for publicly available datasets
- A description of any restrictions on data availability
- For clinical datasets or third party data, please ensure that the statement adheres to our policy

The mass spectrometry proteomics data generated in this study have been deposited to the ProteomeXchange Consortium via the PRIDE partner repository with the dataset identifier PXD041418. Source data are provided with this paper.

Research involving human participants, their data, or biological material

Policy information about studies with human participants or human data. See also policy information about sex, gender (identity/presentation), and sexual orientation and race, ethnicity and racism.

Reporting on sex and gender	n/a
Reporting on race, ethnicity, or other socially relevant groupings	n/a
Population characteristics	n/a
Recruitment	n/a
Ethics oversight	n/a

Note that full information on the approval of the study protocol must also be provided in the manuscript.

Field-specific reporting

Please select the one below that is the best fit for your research. If you are not sure, read the appropriate sections before making your selection.

☒ Life sciences ☐ Behavioural & social sciences ☐ Ecological, evolutionary & environmental sciences

For a reference copy of the document with all sections, see nature.com/documents/nr-reporting-summary-flat.pdf

Life sciences study design

All studies must disclose on these points even when the disclosure is negative.

Sample size	Statistical determination of sample size was not performed. Sample size, statistical tests and P-values are indicated in the methods section or figure legends. Non-quantitative experiments were repeated up to three different times to ensure reproducibility. The sample size in quantitative experiments was decided based on previously published research and to allow a powerful statistical analysis of the data.
Data exclusions	No data were excluded from the analyses.
Replication	Independent biological replica were used in all experiments. All experiments presented in this study were successfully reproduced.
Randomization	Samples were not randomised for this study except for LS-MS data acquisition. Samples were allocated to their experimental groups based on their genotypes.
Blinding	Blinding was not used for data collection or analysis, as the same researcher collected the samples, performed the experiments and analyzed the data. Experimental and control samples were always analyzed in parallel and were treated in the same manner.

Reporting for specific materials, systems and methods

We require information from authors about some types of materials, experimental systems and methods used in many studies. Here, indicate whether each material, system or method listed is relevant to your study. If you are not sure if a list item applies to your research, read the appropriate section before selecting a response.

Materials & experimental systems

n/a	Involved in the study	
☐	☒	Antibodies
☒	☐	Eukaryotic cell lines
☒	☐	Palaeontology and archaeology
☐	☒	Animals and other organisms
☒	☐	Clinical data
☒	☐	Dual use research of concern
☒	☐	Plants

Methods

n/a	Involved in the study	
☒	☐	ChIP-seq
☒	☐	Flow cytometry
☒	☐	MRI-based neuroimaging

Antibodies

Antibodies used	Anti-ATP5H (abcam, ab110275, GR3256550-8)<br>Anti-ATP5A (abcam, ab14748, 2101031804)<br>Anti-NDUFS3 (abcam, ab14711, GR3347771-1)<br>Anti-Beta Actin (Cell Signaling Technology,4967)<br>GABARAP (Cell Signaling Technology ,13733, 13733S)<br>Secondary antibodies:<br>Goat anti- Mouse IgG, antibody Alexa Fluor 448 (Invitrogeen, A11017, 2454774)<br>Goat anti- Mouse IgG, antibody Alexa Fluor 647 (Invitrogeen, A32728,RJ243423)<br>Goat anti-Rabbit, antibody Alexa Fluor 568 (Invitrogen, A11011, 2088069)<br>Mouse IgG HRP (Cytiva, NA9310V, 17925908)<br>Rabbit IgG HRP (Cytiva, NA9340V, 18020658)
Validation	The remaining antibodies have been validated by the manufacturer, validation details and relevant publications are detailed in their respective websites.<br>Anti-ATP5H: abcam, https://www.abcam.com/products/primary-antibodies/atp5h-antibody-7f9bg1-ab110275.html<br>Anti-ATP5A: abcam, https://www.abcam.com/products/primary-antibodies/atp5a-antibody-15h4c4-mitochondrial-markerab14748.html<br>Anti-NDUFS3: abcam, https://www.abcam.com/products/primary-antibodies/ndufs3-antibody-17d95-ab14711.html<br>Anti-beta Actin: cell signaling technology, https://www.cellsignal.com/products/primary-antibodies/b-actin-antibody/4967<br>GABARAP: cell signaling technology, https://www.cellsignal.com/products/primary-antibodies/gabarap-e1j4e-rabbitmab/13733<br>Goat anti- Mouse IgG,Alexa Fluor 448: Invitrogeen, https://www.thermofisher.com/antibody/product/Goat-anti-Mouse-IgGH-L-Cross-Adsorbed-Secondary-Antibody-Polyclonal/A-11001<br>Goat anti- Mouse IgG, Alexa Fluor 647 : Invitrogeen, https://www.thermofisher.com/antibody/product/Goat-anti-Mouse-IgG-H-L-Highly-Cross-Adsorbed-Secondary-Antibody- Polyclonal/A32728<br>Goat anti-Rabbit, Alexa Fluor 568 : Invitrogen, https://www.thermofisher.com/antibody/product/Goat-anti-Rabbit-IgG-H-LCross-Adsorbed-Secondary-Antibody-Polyclonal/A-11011<br>Mouse IgG HRP: Cytiva, https://www.cytivalifesciences.com/en/se/shop/protein-analysis/blotting-and-detection/blottingstandards-and-reagents/amersham-ecl-hrp-conjugated-antibodies-p-06260#related-documents<br>Rabbit IgG HRP: Cytiva, https://www.cytivalifesciences.com/en/se/shop/protein-analysis/blotting-and-detection/blottingstandards-and-reagents/amersham-ecl-hrp-conjugated-antibodies-p-06260#related-documents

Animals and other research organisms

Policy information about studies involving animals; ARRIVE guidelines recommended for reporting animal research, and Sex and Gender in Research

Laboratory animals	Drosophila melanogaster, wDah, and w1118 (Bloomington Drosophila stock Depository) were used in this study
Wild animals	n/a
Reporting on sex	Both sexes were used equally
Field-collected samples	n/a
Ethics oversight	n/a

Note that full information on the approval of the study protocol must also be provided in the manuscript.

Plants

Seed stocks	n/a
Novel plant genotypes	n/a
Authentication	n/a
